# PTM encoding: decoding the mechanisms of exercise-induced metabolic memory through spatiotemporal modification networks

**DOI:** 10.3389/fspor.2026.1769113

**Published:** 2026-04-28

**Authors:** Yinghao Shen, Zhujun Mao, Xinru Zhang, Yupeng Yang, Zhidong Shen, Haodong He, Cheng Chen, Junjie Liu

**Affiliations:** 1 Graduate School, Harbin Sport University, Harbin, China; 2 School of Basic Medicine, China Three Gorges University, Yichang, China; 3 First Clinical Medical College, Shenyang Medical College, Shenyang, China; 4 College of Science and Technology, China Three Gorges University, Yichang, China

**Keywords:** AMPK/mTOR, *in situ* mass spectrometry imaging, interorgan communication, metabolic memory, post-translational modification (PTM) encoding, precision exercise prescription, PTM clock, single-cell multiomics

## Abstract

Post-translational modifications (PTMs) form a sophisticated regulatory layer that modulates cellular responses to physiological stimuli, notably exercise-triggered metabolic adaptations like skeletal muscle glucose uptake. Understanding PTM-mediated regulatory logic, namely how several well-characterized PTMs coordinate spatiotemporally to mediate distinct biological functions, has become a critical frontier in decoding metabolic memory mechanisms. Traditional paradigms focused on isolated signaling pathways struggle to account for the intricate network dynamics underlying these adaptations. This review synthesizes recent advances that clarify the role of PTM-mediated regulation in exercise-induced metabolic memory. We emphasize integrating cutting-edge technologies (single-cell multiomics, *in situ* mass spectrometry imaging). These tools enable constructing dynamic PTM profiles with exceptional spatial and temporal resolution. Innovations in fluorescence reporter probes additionally improve monitoring of PTM dynamics *in vivo*. We explore the molecular logic governing hierarchical PTM networks and interorgan communication, highlighting the central regulatory functions of AMPK/mTOR pathways. Additionally, we discuss emerging machine learning-based PTM clock models that provide quantitative frameworks to track metabolic states and advance precision exercise prescriptions. By bridging molecular insights with translational applications, this review offers a holistic view to advance our understanding of exercise-induced metabolic memory and facilitate developing personalized interventions. These conceptual and technological breakthroughs position PTM-mediated regulation as a transformative paradigm with notable academic value and clinical promise.

## 1 Introduction

Metabolic memory induced by exercise represents a critical concept for understanding physiological adaptations to physical activity and developing strategies to prevent and manage metabolic diseases. Traditional research on exercise-induced metabolic memory has predominantly focused on isolated signaling pathways such as AMPK, mTOR or individual molecular events. While informative, these studies fail to fully capture the complexity and dynamic nature of the involved regulatory networks (1–3). This limited perspective thus hampers a comprehensive understanding of how transient exercise stimuli translate into long-lasting metabolic adaptations (4). In reality, the intricate interplay of multiple molecular signals, their precise temporal and spatial coordination, and integration across cellular and systemic levels necessitate a more holistic approach. Protein PTMs emerge as central to this endeavor, as they can rapidly and reversibly modulate protein function, localization, and interactions in response to environmental cues like exercise (5–7). The dynamic and diverse properties of PTMs provide a molecular substrate for mediating metabolic memory, enabling cells to “remember” prior stimuli and adjust their responses accordingly. Beyond the traditional binary framework of signaling pathways and functional outcomes, the concept of “PTM-mediated regulation” posits that combinations of PTMs act as dynamic information carriers. Interpreted within spatial and temporal contexts, these modifications form a sophisticated molecular regulatory system that orchestrates cellular functions underlying metabolic memory (8–10). Recent advances in technologies including single-cell multi-omics and *in situ* mass spectrometry imaging have greatly enhanced the resolution of PTM dynamics research. They allow real-time monitoring of PTM patterns and their spatial distribution within tissues (11). Furthermore, the identification of inter-organ PTM communication and hierarchical network regulation further elucidates the systemic nature of metabolic memory (12, 13). Integrating machine learning approaches to model PTM “clocks” and leveraging this knowledge to design precision exercise prescriptions hold promise for translating molecular insights into clinical interventions (14). Thus, decoding the PTM landscape in exercise-induced metabolic memory offers a transformative framework to understand and harness metabolism's adaptive capacity for health promotion and disease prevention (15–17).

Exercise exerts significant effects on metabolic regulation and cognitive function, as evidenced by a large body of recent studies employing animal models and human cohorts. In neurodegenerative disease models such as Alzheimer's disease (AD), both high-intensity interval training (HIIT) and moderate-intensity continuous training (MICT) alleviate behavioral deficits like spatial memory impairment and modulate brain metabolism in a region- and exercise-specific manner (18–20). Metabolomic analyses reveal significant changes in energy metabolism indicators, neurotransmitter cycling, and membrane lipid composition across key brain regions including the hippocampus, cortex, and hypothalamus following different exercise modalities (21–23). Notably, HIIT uniquely regulates astrocyte-neuron metabolic coupling, highlighting the nuanced regulatory effects of exercise intensity on cellular metabolic networks (19). Moreover, moderate aerobic exercise enhances hypothalamic insulin-like growth factor 1 (IGF1) signaling, upregulates brain-derived neurotrophic factor (BDNF) expression, and increases glucose transporter 4 (GLUT4) levels. These changes collectively improve spatial memory and reduce amyloid plaque burden in AD models (24). These findings underscore exercise's capacity to orchestrate complex metabolic and neurotrophic pathways that underpin cognitive resilience. Beyond neurodegeneration, physical exercise ameliorates metabolic syndrome and associated cognitive impairments. Voluntary wheel running, for instance, restores peripheral glucose control and normalizes gene expression profiles related to energy metabolism in the brain (25). At the molecular level, exercise-induced modulation of lipid metabolism including enhanced cholesterol transport and reduced soluble amyloid-beta species contributes to neuroprotection and cognitive improvement (26). Mechanistically, exercise promotes autophagy-lysosomal function, facilitating proteostasis and clearance of pathological protein aggregates. This process is essential for maintaining neuronal health and delaying neurodegenerative processes (27). Collectively, these studies highlight the multifaceted metabolic adaptations elicited by exercise, implicating coordinated PTM networks as potential molecular mediators of these systemic benefits.

Protein PTMs are a core regulatory mechanism through which cells dynamically adjust protein function in response to internal and external stimuli, including exercise-induced metabolic stress. PTMs such as phosphorylation, ubiquitination, acetylation, crotonylation and lactylation are several well-characterized types that modulate protein activity, stability, interactions, and localization, thereby transmitting complex biological regulatory information beyond the genetic code. The “PTM-mediated regulation” paradigm conceptualizes these modifications as dynamic information streams that, when integrated with spatial and temporal context, form a regulatory system governing cellular responses. For instance, lysine crotonylation of metabolic enzymes like pyruvate dehydrogenase enhances mitochondrial energy metabolism efficiency, promoting neuronal activation and memory formation (28). Similarly, lactylation of amyloid precursor protein (APP) at lysine 612 regulates the amyloidogenic processing of APP, thereby ameliorating cognitive decline in Alzheimer's disease models. This illustrates how PTMs influence disease-relevant metabolic pathways (29). The ubiquitin system exemplifies the complexity of PTM networks: diverse chain topologies and crosstalk with phosphorylation and acetylation regulate DNA repair, metabolic reprogramming, and cellular stress responses, presenting both challenges and opportunities for therapeutic targeting (30). Histone PTMs, including methylation and acetylation, constitute an epigenetic “histone code” that modulates chromatin structure and gene expression, thereby contributing to metabolic memory and adaptation (31). Advanced mass spectrometry and computational approaches have facilitated high-resolution mapping of PTMs, enabling dissection of their combinatorial patterns and regulatory enzymes (32, 33). These insights establish PTMs as a versatile and dynamic regulatory system integral to metabolic regulation and memory formation in response to exercise stimuli.

Recent technological breakthroughs have revolutionized the study of PTM dynamics, enabling extremely high resolution of their temporal and spatial patterns in physiological contexts such as exercise-induced metabolic memory. Single-cell multi-omics approaches integrate transcriptomic, proteomic, and PTM data at the individual cell level, revealing cellular heterogeneity and cell-type-specific regulatory mechanisms underlying metabolic adaptations. *in situ* mass spectrometry imaging allows direct visualization of PTM distributions within tissue architecture, preserving critical spatial localization information for understanding intercellular communication and organ-specific responses (34). Fluorescent reporter probes have been developed to monitor PTM states in real time, providing dynamic insights into signaling cascades during and after exercise interventions (35). Additionally, engineered 3D human organoids derived from induced pluripotent stem cells (iPSCs) offer physiologically relevant models to study exercise-induced epigenetic and PTM changes in muscle and neural tissues. They help bridge the research gap between animal models and human physiology. Integration of machine learning algorithms facilitates the construction of PTM “clocks” that model the temporal progression of metabolic memory and predict individual responses to exercise regimens, advancing precision medicine applications. These innovative methodologies collectively enable comprehensive decoding of the PTM landscape, fostering mechanistic understanding and translational potential in exercise biology.

The systemic regulation of exercise-induced metabolic memory extends beyond individual cells to rely on inter-organ communication mediated by PTM networks. Skeletal muscle acts as an endocrine organ, releasing myokines such as cathepsin B. These myokines influence brain function by regulating neurogenesis and cognitive-related processes, linking peripheral metabolic adaptations to central nervous system benefits (36, 37). The hypothalamus-skeletal muscle axis exemplifies neuroendocrine crosstalk, where exercise-induced signals coordinate energy metabolism and systemic homeostasis (38). PTM-mediated modifications regulate the secretion and activity of these signaling molecules, orchestrating metabolic adjustments across tissues. Moreover, PTMs modulate the function of transcription factors like peroxisome proliferator-activated receptor alpha (PPAR*α*) in the hippocampus. This modulation is critical for exercise-induced neuroprotection and cognitive improvement in Alzheimer's disease models (39, 40). The integration of PTM networks at multiple hierarchical levels underscores the complexity of metabolic memory as a system-wide phenomenon. Machine learning-driven models of PTM dynamics offer the potential to capture these multilayered interactions, enabling the development of personalized exercise prescriptions that optimize metabolic health and disease prevention. Such precision approaches hold promise for clinical translation, harnessing the molecular underpinnings of metabolic memory to improve healthspan and mitigate metabolic disorders (41–43).

The integration of PTM data with other omics layers—including genomics, epigenomics, transcriptomics, and metabolomics—is essential for constructing comprehensive maps of exercise-induced metabolic adaptations. Recent reviews highlight how multi-omic approaches, combining transcriptomic and epigenetic data with proteomic and phosphoproteomic analyses, provide unprecedented insights into skeletal muscle adaptation to exercise across the lifespan (44, 45). These integrated frameworks reveal that PTM modifications do not operate in isolation but function within complex regulatory networks involving DNA methylation, chromatin accessibility, and gene expression changes. A particularly valuable resource for the exercise research community is the Molecular Transducers of Physical Activity Consortium (MoTrPAC) dataset. This NIH Common Fund initiative has generated large-scale, carefully controlled multi-omic data from both preclinical (rat) and clinical (human) studies, examining responses to endurance and resistance exercise across multiple time points and tissues (46). The MoTrPAC preclinical studies collected 18 tissues from male and female rats at 7 time points following acute exercise, and after 1, 2, 4, or 8 weeks of endurance training, profiling epigenomics (ATAC-seq, RRBS), transcriptomics (RNA-seq), proteomics, post-translational modifications (phosphoproteome, acetylome, ubiquitylome), and metabolomics/lipidomics (47). These datasets, freely available through the MoTrPAC Data Hub, offer unprecedented opportunities for data mining and integration to elucidate PTM-mediated metabolic memory mechanisms (48).

In summary, exercise-induced metabolic memory is governed by complex PTM-mediated regulatory mechanisms that operate across spatial and temporal dimensions, integrating cellular and systemic responses. The dynamic interplay of diverse PTMs orchestrates metabolic adaptations, neuroprotection, and inter-organ communication essential for health maintenance and disease prevention. Advances in high-resolution analytical technologies and computational modeling have propelled the decoding of these complex PTM networks, revealing novel regulatory paradigms beyond traditional pathway-centric views. Understanding and harnessing PTM-mediated regulation in exercise biology offers significant application potential for developing precision interventions tailored to individual metabolic profiles. Future research integrating multi-omics data, machine learning, and innovative model systems will further elucidate the molecular basis of metabolic memory, paving the way for targeted therapies and optimized personalized exercise regimens that enhance metabolic resilience and cognitive function.

## 2 The PTM code: a regulatory grammar for exercise-induced metabolic memory

### 2.1 PTM encoding concept and its biological significance

PTMs represent multiple chemical changes to proteins after synthesis. These include phosphorylation, lactylation, ubiquitination, methylation, acetylation and others. Specifically, the PTM-mediated regulatory encoding mechanism refers to the integration of these discrete modifications into information-rich regulatory patterns that modulate cellular functions. PTMs are not isolated events. They form complex regulatory networks through combinatorial effects and temporal coordination. For instance, phosphorylation can rapidly alter protein activity or intermolecular interactions thereby initiating downstream signal transduction. Exercise type dictates distinct PTM signatures. HIIT and sprinting produce high lactate (5–10 mM), inducing robust histone lactylation (H3K18la) that peaks at 24 h and persists 48–72 h post-exercise—creating a “lactate clock” where tissue modifications outlast circulating lactate clearance (49, 50). Conversely, aerobic exercise generates minimal lactate (∼1 mM) and favors AMPK phosphorylation and mitochondrial acetylation via PGC-1*α* (5). Resistance training presents a paradox: despite high lactate, muscle lactylation remains unchanged due to competition with acetylation for shared lysine residues (51). These modality-specific patterns enable precision exercise prescription for distinct metabolic outcomes (6). Lactylation, a PTM discovered in recent years, is induced by lactate stimulation. It regulates gene expression and metabolic activity highlighting the dynamic link between metabolic status and epigenetic regulation (52) (Table 1).

**Table 1:** PTM effects of different exercise modalities.

Exercise Modality	Core PTM Types	Main Physiological Effects	References
Endurance Exercise (Long-distance Running)	Acetylation, Phosphorylation	Enhanced mitochondrial biogenesis, improved oxidative metabolism	(13, 16)
Resistance Training (Strength Training)	Phosphorylation, Ubiquitination	Activated muscle protein synthesis, muscle hypertrophy	(12, 33)
High-Intensity Interval Training (HIIT)	Lactylation, Acetylation	Metabolic stress adaptation, glycolipid metabolic reprogramming	(6, 52)
Ball Sports (Complex Modality)	Phosphorylation+Ubiquitination+Lactylation (synergistic)	Neuromuscular coordination, improved multi-dimensional exercise performance	(6, 53)

This table shows key exercise-induced PTMs and their physiological pay-offs. Core, reversible marks (acetylation, phosphorylation, etc.) gate signalling pathways unique to each modality; downstream effects improve performance and metabolic control. In complex sports, overlapping PTMs jointly tune neuromuscular coordination, energy supply and inflammation to meet mixed demands.

. The combinatorial patterns of PTMs are often referred to as types such as histone regulatory patterns. Specific combinations of modifications on histone tails regulate chromatin structure and gene transcription in spatial and temporal contexts (31). This combinatorial logic enables cells to produce specific biological effects through a limited set of modification types. The spatial localization of PTMs within cellular subcompartments or chromatin domains further optimizes their functional output. This ensures that signal transduction and metabolic responses are context-dependent. Notably, the PTM-mediated regulatory encoding mechanism breaks through the limitations of traditional linear signaling pathways. It provides a multidimensional framework that captures the complexity of cellular regulation. This theoretical advancement facilitates a deeper understanding of how cells integrate environmental signals, metabolic changes and gene expression programs. Ultimately, it participates in physiological processes such as inflammation, immune response and tissue remodeling (53). By regarding PTMs as an integrated regulatory system, researchers can better decipher the molecular mechanisms underlying cellular memory and adaptive responses. These include responses induced by exercise and metabolic stress.

Table 1. Core Characteristics and Research Basis of Major PTM Types.

This table summarizes the core functions, exercise-related application scenarios, and supporting references of five well-characterized PTM types. It provides a foundational framework for understanding the molecular basis of PTM encoding in exercise-induced metabolic memory.

### 2.2 Establishment of modification grammar rule models

**Figure 1:**
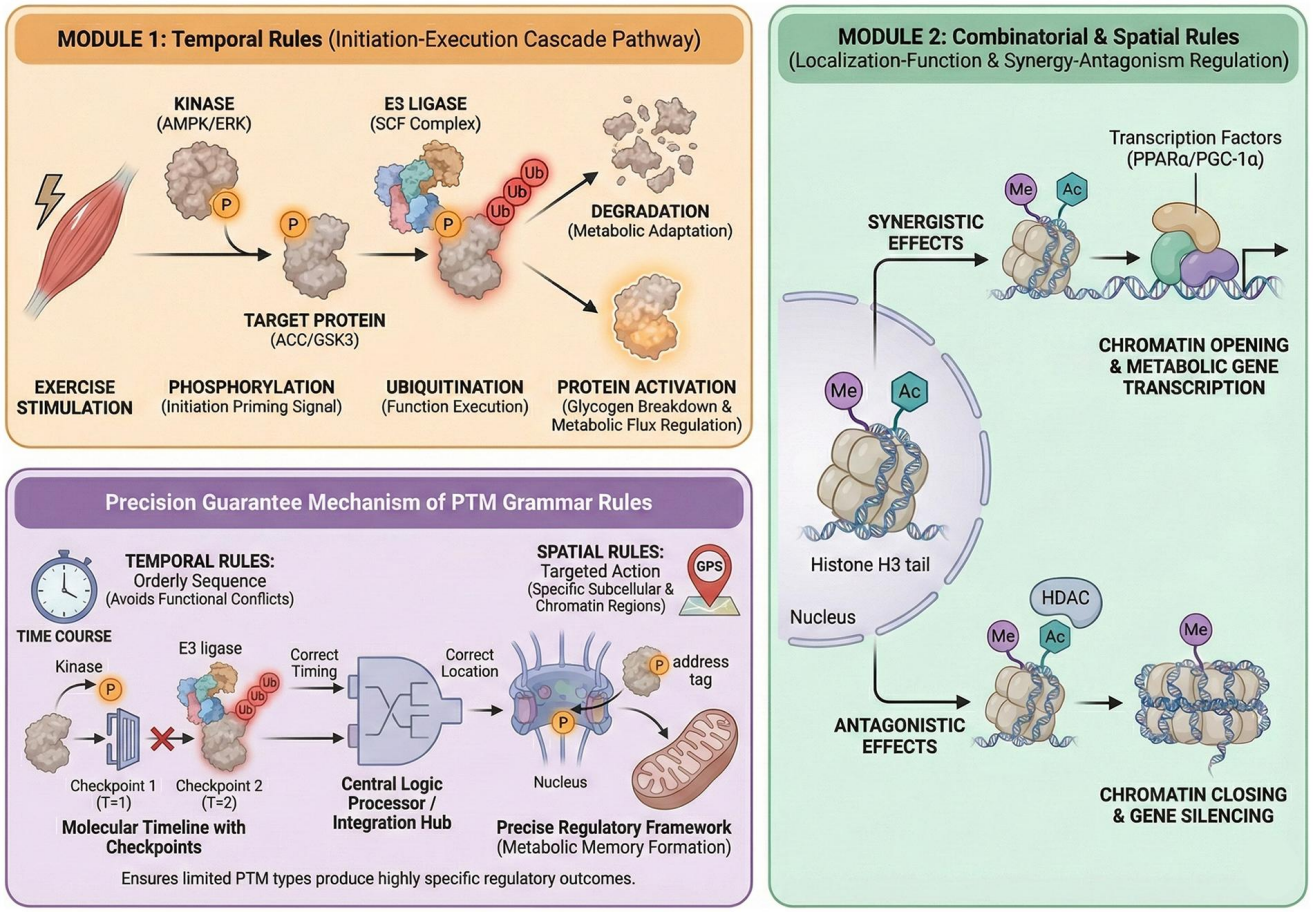
PTM “Grammar Rules” dual-module mechanism diagram. This figure illustrates the core grammar rules of PTM modifications: Temporally, phosphorylation acts as an “initiation signal” to guide the subsequent functional execution of ubiquitination; spatially, methylation and acetylation of histone tails regulate chromatin accessibility through synergistic/antagonistic effects. Together, they ensure that the PTM network precisely regulates metabolism-related protein functions and gene expression under exercise stimulation, representing the key regulatory logic for the formation of metabolic memory.

The “modification grammar rule” model formalizes the combinatorial logic and regulatory rules underlying PTM interactions. This model clarifies the combination methods, temporal sequences of different PTM types and their regulatory effects on protein functions and cellular metabolism. For instance, methylation and acetylation on histone residues can act synergistically or antagonistically. They regulate transcriptional outcomes by influencing chromatin accessibility (31). The temporal sequence of PTMs is crucial. Phosphorylation events may lay the foundation for subsequent ubiquitination of proteins. This process determines the stability or activity state of proteins. Such temporal dynamic characteristics are indispensable for regulating metabolic status and establishing cellular memory. Sequential PTM events can maintain or modulate signaling pathways after the initial stimulus (54). This is particularly critical in metabolic adaptation to exercise. Transient exercise signals need to be encoded into long-lasting metabolic changes. Spatial context also affects the specificity of PTM functions (55). PTMs localized to different cellular subcompartments or nuclear chromatin regions can differentially recruit effector proteins or alter local biochemical environments. In turn, they can finely regulate cellular responses. Molecular mechanisms of spatial specificity include the targeted localization of modifying enzymes to specific loci. It also includes the formation of PTM “hotspot regions” which serve as regulatory hubs. Together, these grammar rules form a predictive framework that can infer how complex PTM patterns translate into specific biological outcomes. It provides new insights for analyzing the regulation of metabolic processes and the formation of metabolic memory (56) (Figure 1).

### 2.3 Application cases of PTM encoding in exercise-induced metabolic memory

Recent studies have confirmed that the PTM-mediated regulatory encoding mechanism can clarify the sustained regulation of metabolic gene expression after exercise. Exercise can induce a series of PTMs in key metabolic organs such as skeletal muscle and liver. These modifications synergistically regulate cellular adaptive responses to increased energy demand. For instance, phosphorylation and lactylation modifications on histones and metabolic enzymes can persist for hours to days after exercise. They maintain epigenetic and metabolic states that facilitate enhanced glucose uptake and mitochondrial biogenesis (31, 52, 57). PTMs function as integrators within a broader muscle memory network, cooperating with myonuclear accretion, DNA methylation, and microRNAs (58, 59). Myonuclei gained during training persist through detraining, maintaining transcriptional capacity for faster re-adaptation, while DNA methylation at metabolic gene promoters creates stable epigenetic priming (60, 61). PTMs dynamically modulate these mechanisms—phosphorylating transcription factors, acetylating chromatin, and linking metabolic flux to epigenetic state—serving as the molecular bridge between acute exercise and long-term adaptation (62, 63). In skeletal muscle, the combinatorial patterns of PTMs regulate the transcription of genes related to oxidative metabolism and muscle remodeling. This process encodes a “memory” of previous exercise which can improve metabolic efficiency during subsequent exercise. In the liver, PTM regulatory networks can modulate gluconeogenesis and lipid metabolism pathways. This helps maintain systemic metabolic homeostasis. These PTM-mediated regulatory mechanisms go beyond the understanding of traditional transient signals. They establish a durable molecular imprint that constitutes the core of metabolic memory (64–67). These findings are of great significance for understanding how repeated exercise induces long-term metabolic adaptations. At the same time, it provides a reference for the development of therapeutic strategies targeting PTM networks which is expected to be used to combat metabolic diseases. The integrated application of the PTM-mediated regulatory encoding mechanism provides a powerful tool for deciphering the molecular basis of exercise-induced metabolic memory across multiple organ systems (68, 69).

## 3 Technical innovations and applications

### 3.1 Single-cell multi-omics and *in situ* mass spectrometry imaging technologies

Single-cell multi-omics technologies have revolutionized the understanding of cellular heterogeneity. They enable simultaneous profiling of multiple molecular layers such as genomics, transcriptomics, proteomics and PTMs at the resolution of individual cells. These approaches prove particularly effective for dissecting the heterogeneity of PTM responses induced by exercise stimuli. Such responses are often masked in bulk analyses. Integrated multi-omics studies in cancer have revealed that PTM pathways are dysregulated in a cell-type-specific manner. Single-cell transcriptomics uncovers differential expression of phosphorylation-related genes like AKT1 in distinct cellular subpopulations (70, 71). Extending these principles to exercise biology, single-cell multi-omics can resolve the heterogeneity of PTM signaling networks across diverse muscle and metabolic cell types. It elucidates how exercise induces specific PTM patterns that encode metabolic memory.

Complementing single-cell multi-omics, *in situ* mass spectrometry imaging (MSI) technologies provide spatially resolved molecular maps of PTMs directly within tissue sections. Techniques such as matrix-assisted laser desorption/ionization (MALDI)-MSI have been adapted to visualize PTMs like glycosylation and phosphorylation with high spatial fidelity. They enable dynamic monitoring of phosphorylation spatial distributions in skeletal muscle fibers within 24 h post-exercise (72, 73). Recent advances include multiplexed imaging mass spectrometry approaches. These employ serial enzyme digests to characterize complex extracellular matrix PTMs within formalin-fixed paraffin-embedded tissues. They preserve spatial context while enhancing PTM coverage (72). Furthermore, integration of spatial transcriptomics with MSI has facilitated the correlation of PTM regulatory patterns with gene expression profiles. It further refines the understanding of PTM-mediated regulatory networks *in situ* (70) (Table 2).

**Table 2:** Interaction between PTM genetic background and exercise responsiveness.

PTM-Related Gene Type	Impact of Genetic Polymorphism	Changes in PTM and Exercise Responsiveness	References
Acetyltransferase genes (ACLY)	Gain-of-function polymorphism	Increased acetylation level; enhanced mitochondrial biogenesis and metabolic improvement post-endurance exercise (high responder phenotype)	(7, 74)
Ubiquitin ligase genes (Parkin)	Loss-of-function polymorphism	Reduced ubiquitination efficiency; delayed muscle repair and weakened hypertrophy effect after resistance training (low responder phenotype)	(20, 66)
Lactylation-related genes (p300)	Upregulated expression polymorphism	Enhanced lactylation sensitivity; faster activation of metabolic genes and shortened recovery cycle post-HIIT (high adaptation phenotype)	(6, 52)
Deacetylase genes (SIRT3)	Loss-of-function polymorphism	Abnormally elevated acetylation level; accumulation of oxidative stress and delayed fatigue recovery post-exercise (exercise intolerance phenotype)	(75, 76)

This table shows the links between polymorphisms in PTM-related genes, changes in PTM, and exercise response phenotypes. It shows the molecular basis for why different people respond differently to exercise.

The combination of single-cell multi-omics and *in situ* MSI offers synergistic advantages for constructing dynamic PTM atlases. Single-cell multi-omics provides high-dimensional molecular profiles with cellular resolution. MSI preserves spatial architecture and heterogeneity within tissues. This integrated approach enables the reconstruction of spatiotemporal PTM networks that underpin exercise-induced metabolic adaptations and memory. However, challenges remain which include the need for improved sensitivity to detect low-abundance PTMs like histone crotonylation at single-cell resolution, enhanced multiplexing capacity and sophisticated computational frameworks for data integration and visualization (74, 77). Addressing these challenges will be critical for leveraging these technologies to decode the complex PTM heterogeneity elicited by exercise. It will also help identify novel biomarkers and therapeutic targets within metabolic tissues.

Table 2: Key Features and Research Basis of Core PTM Research Technologies.

This table compares the core advantages, application scenarios, and relevant references of two cutting-edge technologies. It highlights their synergistic value in resolving the spatiotemporal dynamics of PTM networks during exercise-induced metabolic adaptation.

### 3.2 Spatiotemporal resolution bottlenecks and fluorescent reporter probe technologies

Despite significant advances, current technologies face critical bottlenecks in achieving high spatiotemporal resolution for monitoring PTMs in living systems. Mass spectrometry-based methods, while comprehensive, typically lack real-time temporal resolution. They are limited in spatial precision due to tissue homogenization or limited imaging resolution (78–80). Conversely, fluorescence-based imaging techniques offer superior temporal and spatial resolution. They often suffer from limited multiplexing and specificity for PTM detection. These limitations constrain the ability to capture the dynamic and localized nature of PTM modifications during exercise-induced metabolic remodeling (75, 76).

To overcome these constraints, fluorescent reporter probes specifically genetically encoded PTM biosensors have emerged as powerful tools for real-time, live-cell monitoring of PTM dynamics with subcellular resolution. These probes typically consist of PTM-recognition domains fused to fluorescent proteins or fluorophores. They enable fluorescence resonance energy transfer (FRET) or fluorescence lifetime imaging microscopy (FLIM) readouts that reflect PTM status (81, 82). Kinase activity reporters based on FLIM have demonstrated the capacity to monitor AKT and ERK phosphorylation dynamically in primary muscle cells. They provide insights into signaling kinetics within 5–10 min of exercise onset and subcellular spatial distribution. Similarly, probes targeting acetylation, methylation and ubiquitination have been developed using specific binding domains or engineered recognition motifs (Table 3). They allow multiplexed and reversible detection of PTMs (83, 84).

**Table 3:** Types and applications of fluorescent PTM biosensors.

PTM Target Type	Probe Design Principle	Core Application Scenarios	References
Phosphorylation	FLIM-based kinase activity reporters	Real-time monitoring of AKT/ERK phosphorylation in muscle cells post-exercise	(67)
Acetylation/Methylation	Specific binding domains fused to fluorescent proteins	Multiplexed detection of histone acetylation in live neurons	(69, 70)
Ubiquitination	Engineered recognition motifs+FRET readout	Dynamic tracking of protein ubiquitination during exercise-induced muscle remodeling	(70)

This table outlines the design principles, core application scenarios, and supporting references of fluorescent PTM biosensors. It highlights their advantages in resolving spatiotemporal bottlenecks of PTM detection.

The application of fluorescent PTM biosensors in exercise physiology holds great potential to elucidate the temporal sequence and spatial compartmentalization of PTM signaling cascades that encode metabolic memory (85–88). Live-cell imaging of phosphorylation dynamics in muscle cells during contraction could reveal transient signaling events critical for metabolic adaptation. However, challenges remain in probe design. These include achieving high specificity for PTM types and sites such as AMPK Thr172 phosphorylation, minimizing perturbation of endogenous signaling and expanding multiplexing capabilities to monitor multiple PTMs simultaneously (89, 90). Advances in probe engineering, such as the incorporation of novel fluorophores, improved binding domains and genetically encoded noncanonical amino acids, are addressing these limitations. They are enhancing the sensitivity and versatility of PTM biosensors (83, 91).

Overall, fluorescent reporter probe technologies represent a promising avenue to surmount the spatiotemporal resolution bottlenecks inherent in PTM research. They enable dynamic and spatially resolved interrogation of PTM networks in living cells and tissues during exercise-induced metabolic processes.

Table 3: Types and Applications of Fluorescent PTM Biosensors.

This table outlines the design principles, core application scenarios, and supporting references of fluorescent PTM biosensors. It highlights their advantages in resolving spatiotemporal bottlenecks of PTM detection.

### 3.3 Integration of multi-modal technologies to advance PTM network analysis

The complexity of PTM regulatory networks, characterized by extensive crosstalk between phosphorylation and acetylation and context-dependent regulation, necessitates integrative strategies that combine multi-omics data with advanced imaging modalities. Integrating proteomics, transcriptomics, epigenomics and metabolomics datasets with spatially resolved PTM imaging can yield comprehensive maps of PTM networks. It elucidates their roles in exercise-induced metabolic memory (92, 93). Such multi-modal integration facilitates the identification of key regulatory nodes, PTM crosstalk and dynamic signaling pathways that are otherwise obscured in single-modality analyses.

Data integration strategies leveraging machine learning and artificial intelligence (AI) have been instrumental in decoding PTM spatiotemporal networks. Machine learning algorithms, including random forests, support vector machines and deep learning models, have been successfully applied to classify PTM signatures, predict PTM sites and infer enzyme-substrate relationships from large-scale datasets (11, 70). AI-driven multi-omics integration has enabled the identification of PTM-associated molecular subtypes in cancer. It enhances precision medicine approaches (94). In the context of exercise metabolism, similar approaches can be employed to integrate heterogeneous datasets. They reveal PTM-mediated regulatory circuits underlying metabolic adaptations.

Furthermore, computational tools such as PTMOracle and PTM-POSE facilitate visualization and functional analysis of PTM data within protein interaction networks and splice isoforms, respectively. They enable the exploration of PTM crosstalk and isoform-specific modifications (95, 96). The development of user-friendly platforms like piNET supports downstream annotation and visualization of proteomics data, including PTMs. It accelerates hypothesis generation and validation (97).

Looking forward, the convergence of multi-omics technologies, high-resolution imaging and AI-driven analytics promises to transform the understanding of PTM networks in exercise biology (98). Continued technological advancements will improve throughput, sensitivity and resolution. Integrative computational frameworks will enable the construction of dynamic, spatially resolved PTM networks. These developments will provide unprecedented insights into the molecular encoding of metabolic memory by PTMs. They will ultimately inform targeted interventions to optimize exercise benefits and metabolic health (11).

## 4 Mechanistic research deepening

### 4.1 Inter-organ PTM communication paradigm

**Figure 2:**
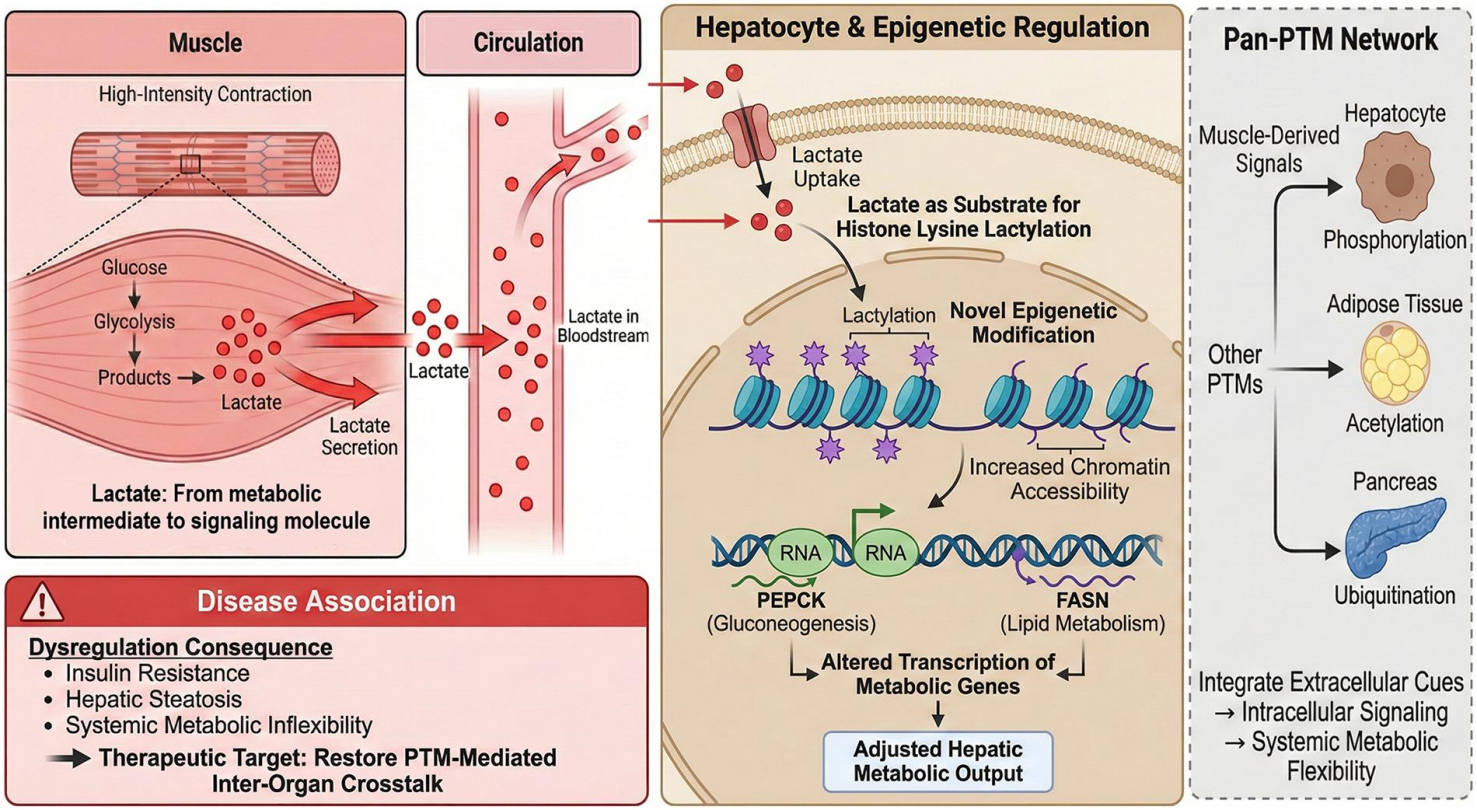
Inter-Organ PTM communication mechanism diagram. This figure illustrates the core pathway of exercise-induced inter-organ PTM communication between muscles and the liver: Exercise triggers skeletal muscle contraction to produce lactate, which is transported to the liver via the blood and taken up by hepatocytes. In the nucleus, lactate acts as a substrate to catalyze histone lysine lactylation (H3K18la), enhancing chromatin accessibility and activating the transcription of target genes related to gluconeogenesis (PEPCK) and lipid metabolism (FASN), ultimately regulating hepatic metabolic output to maintain systemic homeostasis. The dashed line represents the pathological correlation when modification is dysregulated, reflecting the potential role of this PTM communication pathway in metabolic diseases.

The paradigm of inter-organ communication mediated by PTMs has emerged as a focal point for understanding how metabolic tissues coordinate systemic homeostasis (99). A prominent instance is muscle-derived lactate previously viewed as a simple metabolic byproduct. It now acts as a signaling molecule regulating gene expression in distant organs like the liver (100). Recent studies have clarified that lactate produced during high-intensity muscle contraction induces histone lactylation in hepatic cells. This modification modulates transcription of PEPCK and FASN, key metabolic genes in gluconeogenesis and lipid metabolism. Histone lactylation represents a novel epigenetic modification linking metabolic flux to chromatin remodeling and gene regulation. It effectively translates transient metabolic states into sustained transcriptional programs (49–102). The molecular mechanism involves lactate acting as a substrate for histone lysine lactylation. This modification alters chromatin accessibility and recruits transcriptional machinery to target loci. It ultimately adjusts hepatic metabolic output in response to muscular activity (103, 104) (Figure 2). Beyond lactate, well-characterized PTMs such as phosphorylation, acetylation and ubiquitination serve as inter-organ signaling mediators. They convey metabolic adaptation signals from muscle to liver, adipose tissue and pancreas. These PTMs function as molecular regulatory nodes integrating extracellular cues with intracellular signaling cascades. They orchestrate systemic metabolic flexibility (105–107). The functional significance of this inter-organ PTM communication is highlighted by its involvement in metabolic diseases. Dysregulation of lactate-mediated histone lactylation or other PTM signals impairs metabolic gene expression. It contributes to insulin resistance, hepatic steatosis and systemic metabolic inflexibility (Table 4). Thus, understanding the detailed mechanisms of PTM-mediated inter-organ crosstalk offers promising avenues for therapeutic interventions. These interventions target metabolic disorders by restoring or modulating these epigenetic and signaling networks (108–111).

**Table 4:** PTM-Mediated inter-organ communication abnormalities in metabolic diseases.

Disease Type	Abnormal PTM Mechanism	Pathological Effects	References
Insulin Resistance	Dysregulated lactate-mediated histone lactylation in liver	Impaired metabolic gene expression, reduced glucose tolerance	(97, 98)
Hepatic Steatosis	Aberrant myokine acetylation/ubiquitination	Disrupted fat decomposition, lipid accumulation in liver	(94, 99)
Alzheimer's Disease	Defective APP lysine lactylation (K612)	Enhanced amyloidogenic processing, cognitive decline	(30, 100)

This table summarizes the abnormal PTM mechanisms, pathological effects, and supporting references of metabolic diseases related to inter-organ PTM communication. It provides potential therapeutic targets for restoring metabolic homeostasis.

Table 4: PTM-Mediated Inter-Organ Communication Abnormalities in Metabolic Diseases.

This table summarizes the abnormal PTM mechanisms, pathological effects, and supporting references of metabolic diseases related to inter-organ PTM communication. It provides potential therapeutic targets for restoring metabolic homeostasis.

### 4.2 Hierarchical regulation mechanisms of PTM networks

Hierarchical control by key metabolic signaling pathways defines PTM network regulation. These pathways include AMP-activated protein kinase (AMPK) and mechanistic target of rapamycin (mTOR) (112, 113). They act as master regulators integrating nutrient availability and cellular energy status via sensing ATP/ADP ratios. They modulate a diverse set of well-characterized PTMs to coordinate metabolic responses at multiple levels. AMPK activation under energy stress triggers phosphorylation cascades. These cascades promote catabolic processes and inhibit anabolic pathways. They often directly modify enzymes and transcription factors via phosphorylation (114–116). For instance, AMPK phosphorylates PGC-1*α* at Thr177/Ser538 to initiate transcriptional activation, while concurrently activating SIRT1 to deacetylate and stabilize PGC-1*α* at chromatin; subsequently, p38 MAPK phosphorylates Thr262/Ser265 to competitively inhibit ubiquitination, conferring protein stability memory. Conversely, mTOR signaling promotes anabolic growth and biosynthesis. It achieves this through phosphorylation and acetylation events enhancing protein synthesis and lipid metabolism. Notably, AMPK and mTOR pathways exhibit synergistic and antagonistic crosstalk among PTMs. Phosphorylation events influence acetylation patterns and ubiquitination status. This creates a complex molecular logic fine-tuning cellular metabolism (117, 118). This crosstalk is not merely additive but involves feedback loops and hierarchical layering. Upstream kinase activity dictates downstream PTM landscapes. It establishes a dynamic equilibrium between metabolic stability and adaptability. For instance, phosphorylation of metabolic enzymes like ACC1 alters their acetylation susceptibility. Furthermore, ubiquitination regulates the turnover of PTM-modifying enzymes such as histone acetyltransferases. This adds further regulatory depth (Table 5). This hierarchical PTM network maintains metabolic homeostasis under fluctuating environmental and physiological conditions. It allows cells to rapidly adapt to energy availability and stress. Dissecting these layered regulatory mechanisms provides critical insights into metabolic disease pathogenesis. It highlights how PTM network dysregulation contributes to such diseases. It also identifies potential targets for modulating these pathways to restore metabolic balance (119, 120).

**Table 5:** PTM crosstalk and hierarchical regulation in AMPK/mTOR networks.

Regulatory Hierarchy	PTM Crosstalk Type	Metabolic Regulatory Effects	References
Upstream Kinase Level	AMPK/mTOR phosphorylation → Modulates PTM enzyme activity	AMPK activation → Phosphorylation of histone acetyltransferases	(102, 105)
Substrate Protein Level	Phosphorylation → Acetylation (susceptibility change)	ACC1 phosphorylation → Altered acetylation status, enhanced fatty acid oxidation	(106, 108)
PTM Enzyme Turnover Level	Ubiquitination → Regulates PTM-modifying enzyme stability	Ubiquitination of histone acetyltransferases → Modulates acetylation landscape	(109, 111)

This table outlines the hierarchical regulation levels and PTM crosstalk types in AMPK/mTOR networks. It clarifies the complex molecular logic underlying metabolic balance and adaptation to exercise.

Table 5: PTM Crosstalk and Hierarchical Regulation in AMPK/mTOR Networks.

This table outlines the hierarchical regulation levels and PTM crosstalk types in AMPK/mTOR networks. It clarifies the complex molecular logic underlying metabolic balance and adaptation to exercise.

### 4.3 PTM regulation and exercise adaptation correlation

The link between PTM regulation and exercise-induced metabolic adaptation is increasingly recognized as foundational. It explains how physical activity confers systemic health benefits. Exercise triggers a cascade of signaling events that remodel metabolic networks. It does so via dynamic PTM changes such as AMPK Thr172 phosphorylation, histone H3 acetylation and histone lysine lactylation (121–124). Experimental data confirm that exercise-induced PTM networks modulate key metabolic pathways in muscle and other organs. They enhance mitochondrial biogenesis via PGC-1*α* activation, glucose uptake through GLUT4 translocation and fatty acid oxidation. For example, phosphorylation events downstream of AMPK activation during exercise promote GLUT4 translocation. They also activate mitochondrial enzymes like pyruvate dehydrogenase. These changes facilitate improved energy utilization (125–127) (Table 6). Concurrently, PTM dysregulations such as impaired AMPK phosphorylation signaling or aberrant histone acetylation patterns have been linked to reduced exercise capacity. They also contribute to metabolic dysfunction. This underscores the importance of precise PTM regulation for optimal metabolic health (128, 129). Moreover, PTM-mediated epigenetic modifications underpin metabolic memory. Transient exercise bouts induce lasting PTM-mediated epigenetic changes. These changes sustain metabolic improvements by regulating gene expression. This mechanistic insight opens new avenues for PTM-targeted strategies in exercise medicine. These strategies aim to enhance muscle function, prevent metabolic diseases and optimize rehabilitation protocols (130). Future research focusing on the spatiotemporal dynamics of PTM networks during exercise will be crucial. It will aid in developing personalized interventions that harness PTM modulation. These interventions maximize exercise benefits and metabolic resilience (131).

**Table 6:** Correlation between exercise-induced PTM modifications and metabolic adaptations.

PTM Modification Type	Target Molecules	Physiological Effects	References
AMPK Thr172 Phosphorylation	GLUT4, PGC-1*α*	Enhances glucose uptake, promotes mitochondrial biogenesis	(116, 118)
Histone H3 Acetylation	Oxidative metabolism-related genes	Improves skeletal muscle aerobic metabolic efficiency	(5, 32)
Histone Lysine Lactylation	Gluconeogenesis-related genes	Maintains post-exercise glucose homeostasis	(45, 91)

This table summarizes the correlations between key exercise-induced PTM modifications, their target molecules, physiological effects, and references. It clarifies the critical role of PTMs in mediating long-term metabolic adaptations and memory formation.

Table 6: Correlation Between Exercise-Induced PTM Modifications and Metabolic Adaptations.

This table summarizes the correlations between key exercise-induced PTM modifications, their target molecules, physiological effects, and references. It clarifies the critical role of PTMs in mediating long-term metabolic adaptations and memory formation.

## 5 Conclusions and perspectives

The establishment of PTM-mediated regulatory coding as a novel theoretical paradigm marks notable progress in understanding the molecular basis of exercise-induced metabolic memory. By systematically integrating spatiotemporal dynamics of several well-characterized PTMs, this framework provides a systematic framework to decipher the complexity of metabolic regulation. From an expert perspective, synthesizing diverse research findings from molecular mechanisms to systemic crosstalk underscores the transformative potential of PTM-mediated coding in bridging fundamental biology with clinical applications.

**Figure 3:**
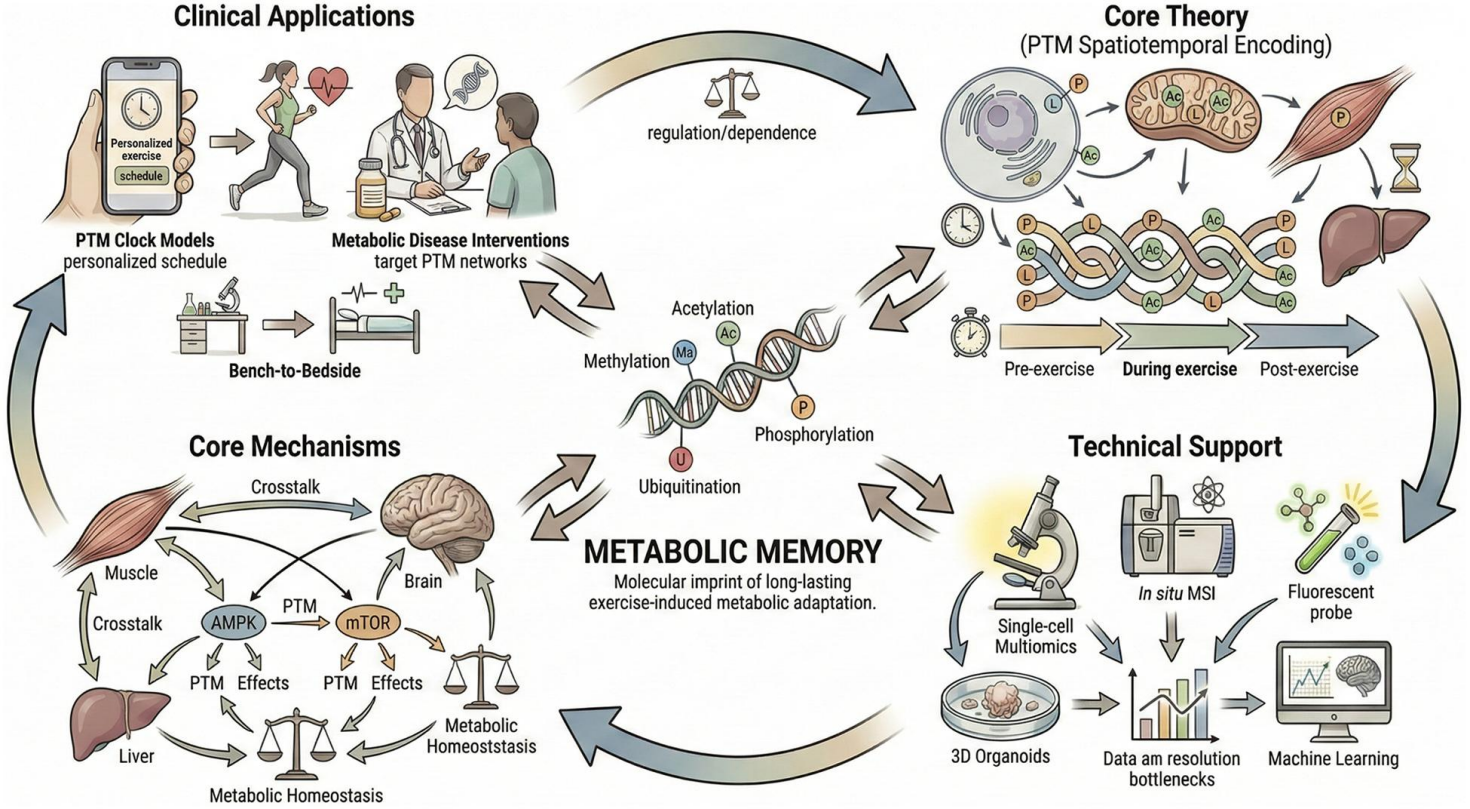
PTM encoding research panorama diagram in exercise-induced metabolic memory. This figure adopts a central-node circular layout to synthesize the core “theory-technology-mechanism-application” framework of PTM (Post-Translational Modification) encoding in exercise-induced metabolic memory. The central node “Metabolic Memory” acts as the core hub, connected to four modules via bidirectional arrows labeled “regulation/dependence.” The four modules form a unidirectional closed loop (Core Theory → Technical Support → Core Mechanisms → Clinical Applications → Core Theory), reflecting the iterative nature of the paradigm. Core Theory establishes the molecular basis, Technical Support provides methodological guarantees including single-cell multi-omics and MSI (Mass Spectrometry Imaging), Core Mechanisms uncovers key regulatory logic involving AMPK (AMP-activated Protein Kinase) and mTOR (mechanistic Target of Rapamycin) pathways, and Clinical Applications realizes “bench-to-bedside” translation. The diagram intuitively reflects the transformative academic and clinical value of PTM encoding as a core paradigm in this field.

Recent technological breakthroughs, particularly single-cell multi-omics and *in situ* mass spectrometry imaging, have been instrumental in constructing high-resolution dynamic PTM profiles (132, 133). These tools enable unprecedented granularity in mapping PTM patterns within skeletal muscle fibers and hepatic cells. They also capture temporal changes across hours to days post-exercise. Complementing these advances, fluorescence reporter probe technologies further enhance our ability to monitor PTM events in real-time with subcellular spatial precision. Together, these methodological innovations have deepened mechanistic insights (Figure 3). They have also laid the groundwork for more nuanced investigations into PTM-mediated metabolic adaptations.

A critical dimension of this evolving field is the elucidation of inter-organ PTM communication and hierarchical network regulation. Notably, discoveries highlighting the central role of AMPK and mTOR signaling pathways within PTM networks have refined our conceptual understanding of metabolic adaptability. These findings emphasize that PTM modifications do not operate in isolation. They function within intricate inter-organ crosstalk between skeletal muscle and the liver and regulatory hierarchies. These elements are essential for maintaining metabolic homeostasis in response to exercise stimuli. Balancing molecular specificity with systemic integration remains a challenging yet necessary endeavor to fully appreciate the complexity of PTM-driven metabolic control.

Construction of PTM clocks requires integration of multi-level spatiotemporal data: dynamic modification profiles, tissue-specific networks, exercise history trajectories, and multi-omics covariates. However, accessibility of deep tissues constitutes a major technical bottleneck. Feasible alternative strategies include: (1) peripheral blood-tissue correlation models—circulating extracellular vesicles carry tissue-specific PTM-characteristic proteins; (2) metabolite surrogate markers—blood lactate/ketone body ratios reflect hepatic histone lactylation levels; (3) wearable FRET sensors for real-time monitoring of skin ATP/ADP ratios to indirectly infer AMPK activity. It must be acknowledged that these surrogate markers have limited tissue specificity. Furthermore, inter-individual baseline PTM heterogeneity—stemming from aging-related NAD^+^-SIRT1 axis decline, sexual dimorphism in acetylation vs. phosphorylation sensitivity, obesity-induced AMPK resistance with lactylation uncoupling, and fitness-level-dependent histone methylation patterns—necessitates dynamically calibrated, stratified models incorporating age-correction, sex-specific network topology, and disease-baseline compensation algorithms, rather than statically thresholded approaches, to achieve translation from population-averaged to individualized precision.Importantly, the integration of machine learning approaches such as random forest and deep learning to develop PTM clock models and the conceptualization of precision exercise intervention frameworks mark a notable transition toward personalized medicine. These innovations harness the predictive power of computational modeling to tailor exercise interventions based on individual PTM signatures. They thereby optimize metabolic health outcomes and disease prevention strategies. This convergence of bioinformatics, systems biology, and clinical science exemplifies the interdisciplinary synergy required to translate PTM research from bench to bedside.Overall, PTM-mediated coding research stands at the forefront of metabolic biology, offering substantial theoretical advancements and practical translational opportunities. It not only deepens our fundamental understanding of metabolic mechanisms but also paves new avenues for precision exercise medicine and metabolic disease management. The academic significance of this field is matched by its promising application potential, marking a new phase where molecular insights into PTMs can inform individualized therapeutic strategies and improve public health outcomes. Continued efforts to harmonize diverse research perspectives, refine technological methodologies, and validate clinical applications will be essential to fully realize the impact of PTM-mediated coding in both scientific and medical domains (134, 135).
